# The effects of male social environment on sperm phenotype and genome integrity

**DOI:** 10.1111/jeb.13435

**Published:** 2019-03-25

**Authors:** Willian T. A. F. Silva, Paula Sáez‐Espinosa, Stéphanie Torijo‐Boix, Alejandro Romero, Caroline Devaux, Mathilde Durieux, María José Gómez‐Torres, Simone Immler

**Affiliations:** ^1^ Department of Evolutionary Biology Uppsala University Uppsala Sweden; ^2^ Department of Biotechnology University of Alicante Alicante Spain; ^3^ School of Biological Sciences University of East Anglia Norwich UK; ^4^ Cátedra Human Fertility University of Alicante Alicante Spain

**Keywords:** DNA damage, sexual selection, sperm competition, trade‐offs

## Abstract

Sperm function and quality are primary determinants of male reproductive performance and hence fitness. The presence of rival males has been shown to affect ejaculate and sperm traits in a wide range of taxa. However, male physiological conditions may not only affect sperm phenotypic traits but also their genetic and epigenetic signatures, affecting the fitness of the resulting offspring. We investigated the effects of male‐male competition on sperm quality using TUNEL assays and geometric morphometrics in the zebrafish, *Danio rerio*. We found that the sperm produced by males exposed to high male–male competition had smaller heads but larger midpiece and flagellum than sperm produced by males under low competition. Head and flagella also appeared less sensitive to the osmotic stress induced by activation with water. In addition, more sperm showed signals of DNA damage in ejaculates of males under high competition. These findings suggest that the presence of a rival male may have positive effects on sperm phenotypic traits but negative effects on sperm DNA integrity. Overall, males facing the presence of rival males may produce faster swimming and more competitive sperm but this may come at a cost for the next generation.

## 1 INTRODUCTION

Sperm quality and performance determine male reproductive success and are therefore under strong selection (Birkhead, 1998; Birkhead, Hosken, & Pitnick, 2008; Fitzpatrick & Lüpold, 2014; Snook, 2005). Nevertheless—or perhaps precisely because of their fundamental role—many of the sperm traits exhibit substantial plasticity. Males tailoring their ejaculates to environmental and social conditions have been subject to intense research ever since Geoff Parker's discovery of sperm competition (Parker, 1970). Parker's theoretical work predicted males to invest more into ejaculate traits, such as sperm number and sperm size (indirectly affecting sperm competitiveness through for example speed) that increase its chance to outcompete rival males in situations of sperm competition (e.g. Parker, 1990; Parker, Ball, Stockley, & Gage, 1997). Those theoretical predictions are supported by a large body of empirical evidence (reviewed in Birkhead & Møller, 1998; Snook, 2005; Birkhead et al., 2008). A general interpretation of these findings is that the observed changes in sperm and ejaculate traits are likely adaptive as they improve a male's chance to increase its fitness by fertilizing a larger proportion of eggs over time.

More generally, variation in male social environment such as the presence of other males or female quality is known to affect ejaculate and sperm traits, including sperm numbers, sperm swimming speed, sperm viability, longevity and morphometry in a wide range of taxa (see Snook, 2005; Fitzpatrick & Lüpold, 2014 for reviews). These same sperm traits have also been shown to be directly linked to male fertilization success. In addition to these well‐studied sperm traits, a number of less well‐understood processes, namely physiological reactions to the fertilization environment that sperm are exposed to after ejaculation, are likely to further determine the fertilization ability of sperm. In particular, sperm efficiency to fertilize eggs may be affected by the conditions in the fertilization environment, which are generally very different from the male reproductive tract due to factors such as osmolality, acidity, temperature and UV light (Birkhead, Møller, & Sutherland, 1993). In external fertilizers, the change in osmolality, for example, is the trigger to activate sperm (Alavi & Cosson, 2005). However, the hypo‐ or hyper‐osmotic pressures can also have negative effects, and sperm of different teleost species react differently to such osmotic stresses (Alavi & Cosson, 2005). Similarly, factors such as the seminal fluid of rival males (Bartlett, Steeves, Gemmell, & Rosengrave, 2017; Locatello, Poli, & Rasotto, 2013) or the ovarian fluid around eggs (Urbach, Folstad, & Rudolfsen, 2005) strongly affect sperm swimming behaviour in externally fertilizing fish. However, we currently know little about the underlying mechanisms that induce these rapid changes in sperm morphology and performance.

In addition, male social environments may have strong effects on male physiological conditions and more than just sperm phenotypes may be altered. The social environment affects the stress levels experienced by individuals in a population (e.g. Sapolsky, 2005; Wingfield & Sapolsky, 2003). These stressors may in turn negatively affect the overall quality of sperm within an ejaculate (Cornwallis & Birkhead, 2007; Pizzari, Cornwallis, & Froman, 2007) due to a possible stress‐induced increase in the production of reactive oxygen species (ROS; Aschbacher et al., 2013; Schiavone, Jaquet, Trabace, & Krause, 2013) and activity of transposable elements (Capy, Gasperi, Biémont, & Bazin, 2000), which may cause mutations during gamete production. These factors may result in DNA alterations and damage ranging from point mutations to unrepaired double‐strand breaks. It is therefore possible that males under stressful conditions may produce sperm with higher rates of DNA damage and mutations, which may negatively affect their offspring. Similar reactions are known to occur during sperm ageing processes before and after ejaculation (Pizzari, Dean, Pacey, Moore, & Bonsall, 2008; Reinhardt, 2007). Measuring sperm quality should therefore involve the assessment of both sperm phenotype and sperm genetic content.

Stress‐induced alterations and damages to the sperm DNA not only decrease the quality of the sperm but may also have deleterious effects on the fitness of the resulting offspring. Despite the well‐documented effects of the male social environment on sperm production, only few studies assessed the possible effects of male–male competition on offspring development and fitness. A study in the whitefish (*Coregonus lavaretus*) showed that subordinate males produced more motile sperm but sired offspring with impaired hatching success (Kekäläinen, Soler, Veentaus, & Huuskonen, 2015). In the yellow dungfly (*Scathophaga stercoraria*), superior sperm competitors sired offspring that hatched relatively faster but offspring survival was not associated with the father's success to outcompete rival males (Hosken, Garner, Tregenza, Wedell, & Ward, 2003). In the zebrafish (*Danio rerio*), males exposed to high male–male competition sired faster hatching offspring but showing reduced survival compared to offspring sired by males exposed to low competition (Zajitschek, Hotzy, Zajitschek, & Immler, 2014). These previous findings suggest that a potential trade‐off might exist between the benefits of producing “more competitive” sperm for the father and the potentially negative effects this may have on the resulting offspring, which motivated our study on zebrafish sperm.

We exposed adult male zebrafish to either a high competition treatment, keeping two males with one female, or a low competition treatment, keeping one male with two females. We collected sperm after 2 weeks of treatment exposure and assessed inactivated and activated sperm for head and midpiece morphometry, flagellum length and conformation and DNA integrity. Our findings indicate a potential trade‐off between sperm phenotypes and the integrity of the sperm genome.

## 2 MATERIALS AND METHODS

### 2.1 Experimental fish

Zebrafish from the AB wild‐type strain were obtained from ZIRC and bred under an outbreeding regime at the SciLifeLab zebrafish facility at Uppsala University. The fish were raised to sexual maturity and kept under standard laboratory conditions at a temperature of 28°C, a 12:12 h light‐dark cycle, and an *ad libitum* feeding regime with live *Artemia* (ZM Systems, UK) and dry food (Medium granular, ZM Systems, UK) three (adult) to five (juvenile) times a day. Prior to exposure to the experimental treatments, fish were kept in 10L tanks containing about 50 fish/tank and a sex ratio of about 1:1. Experiments were carried out in 3L tanks in an automated flow‐through system ensuring a continuous water exchange. The experimental protocols were approved by the Swedish Board of Agriculture (Jordbruksverket, approval no. C 3/15).

### 2.2 Experimental set‐up

We exposed zebrafish males for 2 weeks (this duration is long enough for two spermatogenic cycles to be completed and the treatment effects to be present in mature sperm; Leal et al., 2009) to one of two social treatments previously described in Zajitschek et al. (2014). In short, males were either (a) kept under a low competition regime (one male with two females per tank; *n* = 8), or (b) under a high competition regime (two males with one female per tank; *n *=* *8) (electronic Supporting Information Figure S1a). Because zebrafish can display social dominance based on body size (Spence, Gerlach, Lawrence, & Smith, 2008), experimental fish were selected for similar body sizes in each experimental tank. Artificial aquaria plants were added to the tanks in order to provide sheltering and hiding space to reduce aggression and the potential for injuries. The main reason for keeping the animals in small groups is that zebrafish are shoaling fish and direct contact allows them to behave naturally; keeping them in isolation (even with visual and olfactory contact with other fish) prevents them from shoaling, which would lead to significant stress levels (Parker, Millington, Combe, & Brennan, 2012; Piato et al., 2011) and could jeopardize the outcome of the experiment. In the zebrafish, both males and females compete for spawnings and females may dominate males as well as other females, resulting in similar spawning rates between treatments (if any spawning occurs at all as zebrafish tend not to spawn in standard system tanks due to suboptimal conditions; Spence et al., 2008). This means the likelihood that any differences observed between sperm produced by males exposed to the two treatments are due to different spawning rates is very small.

Sperm were collected from each of the experimental males after 14 days of exposure to one of the two treatments. The duration of treatment exposure was twice as long as one spermatogenic cycle in zebrafish (Leal et al., 2009) to allow a treatment effect to be present in mature sperm (Kustan, Maruska, & Fernald, 2012; Rudolfsen, Figenschou, Folstad, Tveiten, & Figenschou, 2006). For sperm collection, males were first anesthetized in a 0.016% MS‐222 solution (Sigma‐Aldrich, A5040) for a maximum of two minutes, briefly rinsed in system water and placed ventral side up into a moist sponge under a stereomicroscope. A paper towel was used to blot dry the genital pore and avoid unwanted sperm activation upon contact with water. Using a calibrated micro‐capillary (Sigma‐Aldrich, P0674), sperm were collected, immediately mixed with 50 μL of Hank's buffer (recipe described in Westerfield, 2007) in microtubes and placed on ice until further steps were conducted.

Each ejaculate was split into five subsamples of 10 μL each, which were fixed in 2% paraformaldehyde 0 (inactivated), 0.5, 1, 5 and 10 min post‐activation (mpa) with 40 μl of water (1 volume of sperm in Hank's buffer and water to 1 volume of 4% paraformaldehyde). After 1 hr of fixation at 4ºC, samples were centrifuged (350 g, 5 min) to replace paraformaldehyde with PBS and conserved at 4ºC. This procedure was used to observe differences in susceptibility to DNA fragmentation and sperm flagellum conformation before and after activation. Three different methods were used to analyse fixed sperm cells (electronic Supporting Information, Figure S1b): a TUNEL assay was used to assess double‐strand DNA fragmentation, tubulin immunostaining was used to easily observe sperm flagellum conformation, and geometric morphometrics was used to analyse sperm head shape and size.

### 2.3 TUNEL assays

A TUNEL (Terminal deoxynucleotidyl transferase dUTP Nick‐End Labeling) assay was conducted on a subsample of each ejaculate to estimate the proportion of inactivated sperm containing high levels of double‐strand DNA breaks (indicative of apoptotic activity) and the rate at which sperm experience double‐strand DNA breaks after activation. To detect apoptotic cells, 5 μl of each fixed sperm subsample was transferred to round cover slips of 15 mm of diameter, air‐dried, rehydrated three times with PBS (1×) for 5 min each and permeabilized with 0.2% Triton X‐100 for 5 min. After permeabilization, samples were washed in PBS (1×) and labelled using a TdT reaction mix (DeadEnd^™^ Fluorometric TUNEL System, Promega) for 1 hr at 37°C in a dark humidified chamber. Finally, samples were washed three times in PBS (1×) for 5 min and assembled on microscope slides using Vectashield H‐1000 mounting medium with DAPI (Vector Laboratories). Apoptosis was quantified by scoring 200 sperm cells of each subsample using fluorescence microscopy.

### 2.4 Tubulin immunostaining

Sperm flagellum conformation (uncoiled, partially coiled and fully coiled) was used as an indication of the effect of the osmotic stress on sperm activity. Sperm with partially or fully coiled flagella are less agile or completely immobilized. In order to assess sperm flagellum conformation, a tubulin immunostaining protocol was conducted on a subsample of each ejaculate. As for the TUNEL protocol, sperm subsamples were dried, rehydrated and permeabilized. In a next step, subsamples were washed in PBS (1×) and blocked using 2% BSA‐PBS for 30 min. Tubulin was evaluated using a monoclonal anti‐α‐tubulin antibody produced in mouse (Sigma‐Aldrich) at a concentration of 1:600 for 1 hr at room temperature in a dark humidified chamber. Following primary antibody incubation, samples were washed three times in PBS (1×), covered in the secondary antibody against mouse IgG conjugated to Alexa Fluor^®^ 488 (Jackson ImmunoResearch) at a concentration of 1:300 and incubated at room temperature in a dark humidified chamber for one hour. This protocol was based on procedures previously described by Chauvigné, Boj, Finn, & Cerdà, 2015. Finally, samples were washed three times in PBS (1×) for 5 min each and assembled on microscope slides using Vectashield H‐1000 with DAPI (Vector Laboratories). Flagellum conformation patterns were quantified by scoring 200 sperm cells of each subsample using fluorescence microscopy. Sperm cells were classified based on flagellum conformation as fully coiled, partially coiled or uncoiled (electronic Supporting Information, Figure S2). Detailed micrographs of sperm head and flagellum conformation were recorded using scanning electronic microscopy (SEM) (see below).

### 2.5 SEM sample preparation and micrographs

Morphometric data were obtained from sperm at 0 (inactive) and 30 s post‐activation (spa) from six males per treatment. After the primary fixation, samples were washed three times in PBS, centrifuged at 350 g for 5 min and subsequently fixed with 1% osmium tetroxide (Electron Microscopy Sciences, Hatfield, PA, USA) in PBS for 30 min. Sperm were washed again three times by centrifugation and 5 μl were then placed on a glass coverslip and dehydrated in an ethanol series (50%, 70%, 80%, 90%, 96%, and absolute alcohol) and critical point dried in carbon dioxide (EMS850, Electron Microscopy Sciences). All coverslips were glued to the stubs by carbon adhesive tape, then AU sputtered (Balzers SCD 004 Sputter Coater) and examined under a SEM (Hitachi S3000N). High‐resolution SEM micrographs of sperm heads and flagella were recorded at 10,000 × magnification and an accelerating voltage of 10 kV. An average of 20 sperm cells from each male was included in the analysis. Sperm head micrographs were randomly selected and processed on Adobe^®^ Photoshop^®^ CS3 to obtain standardized sperm head orientation images (1024 × 756 TIFF‐file format). For each sperm image, we digitized a set of two‐dimensional anatomical landmark and sliding semilandmark coordinates using tpsDig2 (Rohlf, 2015) to describe sperm morphology based on spatial position of particular anatomical traits. Zebrafish sperm are structured in a head without acrosome, midpiece and flagellum. Four fixed landmarks represent discrete identifiable points at the intersection of the midpiece structure with the sperm head and points of maximum curvature of the midpiece basal contour. Apart from these landmarks, MakeFan6 (Sheets, 2001) was used to register 18 equidistant semilandmarks located across the sperm head periphery based on their centroid position and three additional semilandmarks were placed to register the midpiece structure of each sperm (electronic Supporting Information, Figure S3). In addition, the length of the flagellum of 10 inactivated sperm from each male was measured using ImageJ (NIH).

### 2.6 Data analysis

Effects of social environment and activation status on flagellum length and conformation and double‐strand DNA fragmentation were statistically analysed using generalized linear mixed effects models (function *glmer* in the R package *lme4)* with a binomial error distribution and logit link function including treatment and minutes post‐activation (mpa) as fixed effects and male ID and tank ID as random effects. For the flagellum conformation analysis, the response variable was the number of sperm cells exhibiting uncoiled/partially coiled/fully coiled flagella out of 200 cells counted per sample. For the DNA integrity analysis, the response variable was the number of sperm cells showing strong TUNEL signal out of 200 cells counted per sample. We started with a full model including the interaction terms and removed those if not significant. Treatment effects on flagellum length were analysed using a linear mixed effects model with treatment as fixed effect and male ID and tank ID as random effects.

In order to extract shape information from the landmark coordinates of sperm heads, a Generalised Procrustes Analysis (GPA) superimposition was performed to remove the effects of scale, translation/location and orientation (Dryden & Mardia, 2016). A sliding landmark algorithm was applied to minimize the bending energy (Gunz & Mitteroecker, 2013) using TpsRelw (Rohlf, 2015). Sperm head size was computed as the centroid size (CS), calculated as the square root of the sum of squared distances of all the landmarks from their centroid (Dryden & Mardia, 2016; Klingenberg, 2011). To characterize the effects of size‐related variation in shape (allometry), we used a multivariate regression of sperm shape (Procrustes shape coordinates) on the log‐transformed CS as a predictor variable (Klingenberg, 2016; Monteiro, 1999) of each activation state (0 spa and 30 spa) separately. A permutation test (10,000 permutations per test) was performed for each multivariate regression in order to assess the statistical significance of the association between sperm size and shape (Klingenberg, 2011, 2016). We further tested for size and shape differences by using the regression residuals from the multivariate regressions as response variable in a linear mixed effects model (*lmer)* with male ID as random factor. The Procrustes distances (*D*) between the mean shapes of the sperm by social treatments (high and low sperm competition) were derived from a discriminant function analysis (DFA). Morphometric and statistical analyses were performed in MorphoJ (Klingenberg, 2011) and R version 3.4.3 (R Core Team, 2017). The significance level was set at α*≤*0.05.

## 3 RESULTS

**Figure 1:**
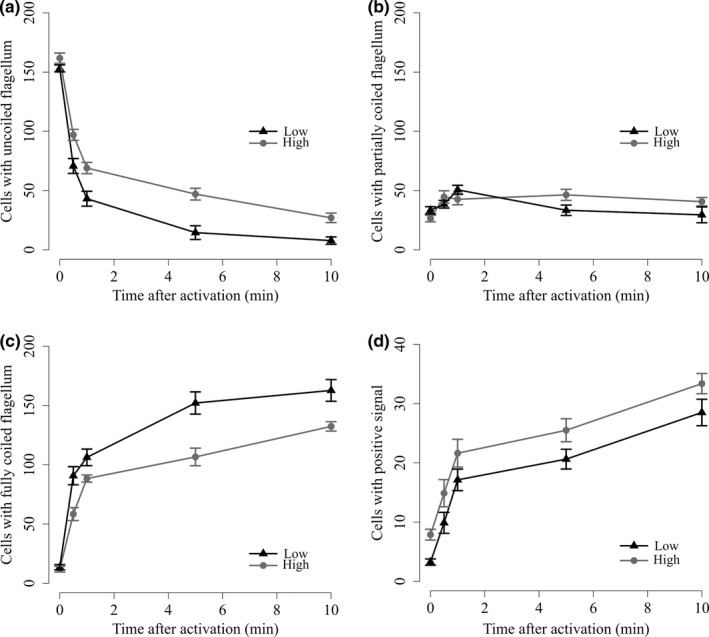
Sperm flagellum conformation and DNA integrity changes between social treatments before and after activation (in minutes; min). Change in number of sperm cells with uncoiled (a), partially coiled (b) and fully coiled (c) flagellum assessed with tubulin immunostaining. (d) Change in number of sperm cells showing a positive signal of double‐strand DNA breaks assessed with TUNEL assay. Exactly 200 sperm for each subsample and assay were assessed, and hence, changes in frequency are relative. Error bars denote ±SE (standard error)

We found significant differences in flagellum length, flagellum conformation, DNA fragmentation and sperm head morphometrics between males exposed to the two different social treatments. Specifically, sperm produced by males from the high competition treatment exhibited a longer flagellum than sperm produced by males in the low competition treatment (*lmer* statistics: $\chi_{1}^{2}$ = 7.09, *p *=* *0.008; high competition: 29.86 ± 2.05 μm (mean ± *SD*); low competition: 28.45 ± 2.58 μm). The number of sperm with uncoiled flagella was high and did not differ between the treatments in inactivated sperm, but males in the high competition treatment produced sperm that retained an uncoiled flagellum for longer post‐activation as indicated by the significant interaction term (*glmer* statistics: treatment: $\chi_{1}^{2}$ = 2.61, *p* = 0.106; spa: $\chi_{1}^{2}$ = 960.04, *p < *0.001; treatment*spa: $\chi_{1}^{2}$ = 146.10, *p *<* *0.001; Figure 1a). Accordingly, the number of partially coiled flagella did not differ significantly between treatments in both, inactivated and activated sperm and did not increase drastically over time post‐activation (treatment: $\chi_{1}^{2}$ = 0.32, *p* = 0.572; mpa: $\chi_{1}^{2}$ = 5.88, *p* = 0.015; treatment*mpa: $\chi_{1}^{2}$ = 27.35, *p *<* *0.001; Figure 1b). The number of sperm with fully coiled flagella in inactivated sperm was low in both treatments and increased with time and at a faster rate post‐activation in sperm produced by males under low sperm competition as indicated by the significant interaction term (treatment: $\chi_{1}^{2}$ = 2.96, *p* = 0.085; mpa: $\chi_{1}^{2}$ = 931.74, *p < *0.001; treatment*mpa: $\chi_{1}^{2}$ = 62.82, *p *<* *0.001; Figure 1c). In contrast, the TUNEL assay showed that males under low competition conditions produced less sperm exhibiting DNA double‐strand breaks in the inactivated state. The amount of cells exhibiting DNA double‐strand breaks increased over time post‐activation but at a similar rate in both treatments as indicated by a nonsignificant interaction term and a persistent difference between treatments over time (treatment: $\chi_{1}^{2}$ = 6.21, *p* = 0.012; mpa: $\chi_{1}^{2}$ = 285.58, *p < *0.001; Figure 1d and electronic Supporting Information Figure S4). These observations indicate that males under low competition seem to produce sperm with a lower level of DNA damage, but with a faster change in flagellum conformation, potentially affecting swimming efficiency.

**Figure 2:**
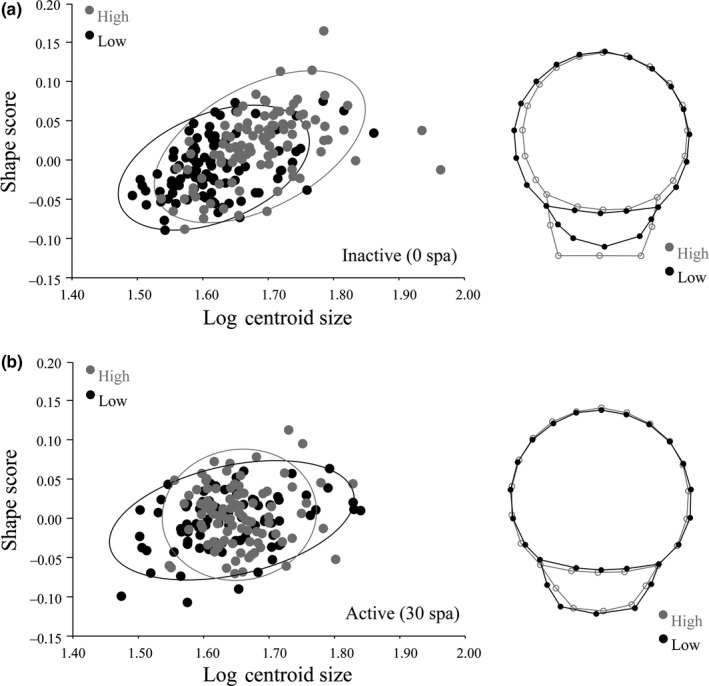
Sperm head shape variation between inactive (a) and active (b) treatments associated with low and high competition. Scatter plots show the shape scores plotted against size (logCS). The ellipses include 95% confidence regions of the competition treatments. Procrustes distances (D) and *p*‐values for DFA: inactive (0 spa): D = 0.033, *p *< 0.001; active (30 spa): D = 0.026, *p *< 0.01. Wireframes denote mean shapes. Note significant sperm shape changes in high competition males

In inactivated sperm (0 spa), we found that sperm shape and size (residual values from a multivariate regression analyses; 7.626% variance explained; *p *<* *0.0001) varied significantly between the two treatments (treatment: $\chi_{1}^{2}$ = 13.83, *p* = 0.0002). Sperm produced by males exposed to high competition exhibited greater size and showed significantly more variation in shape. These sperm were also characterized by a narrower head and a more pronounced midpiece compared to sperm from low competition males (Figure 2a). In contrast, in activated sperm (30 spa), sperm shape and size no longer differed between treatments (treatment: $\chi_{1}^{2}$ = 0.12, *p* = 0.73) and sperm size no longer contributed to the total amount of shape variation (0.985%; *p* = 0.083). Sperm size at 30 spa was similar between social treatments, and sperm head shape variation was reduced among sperm produced by high competition males (Figure 2b).

## 4 DISCUSSION

Our findings show a clear effect of male social environment on sperm phenotypes and DNA quality. However, while most of the previous results suggested that morphological and functional changes in sperm in response to male–male competition may be adaptive and purely beneficial (reviewed in Snook, 2005), our results indicate that individual sperm traits may be affected differently and potentially in opposite directions. We found that sperm phenotypes appear to change in order for sperm to perform better during fertilization (i.e. longer flagellum, slender head and larger midpiece, making sperm faster), but possibly at the cost of reduced genome integrity indicated by the higher amount of sperm exhibiting double‐strand breaks in males exposed to high male–male competition. These findings could indicate that a higher fertilization success benefitting the male may come at the cost of reduced offspring quality (lower DNA quality). Such a hypothesis is also in line with the results in earlier studies, where investment in high‐quality sperm (more competitive sperm) has been associated with lower offspring survival (e.g. Kekäläinen et al., 2015; Zajitschek et al., 2014). We discuss possible underlying mechanisms and the broader implications of our findings.

### 4.1 Morphometry

Male social environments have been shown to affect different morphological sperm traits such as flagellum length (Crean & Marshall, 2008; Immler, Pryke, Birkhead, & Griffith, 2010) and midpiece size (Firman & Simmons, 2010). Our findings in the zebrafish are in line with these previous results in that males in the high competition treatment produced sperm with a more slender head, a larger midpiece and a longer flagellum. These traits are the putative characteristics of faster swimming sperm (Bennison, Hemmings, Brookes, Slate, & Birkhead, 2016; Cardullo & Baltz, 1991; Firman & Simmons, 2010; Mossman, Slate, Humphries, & Birkhead, 2009) and seem to fit our previous observations of faster swimming sperm produced by males in a high male–male competition environment than by males in low competition environments using the same experimental set‐up as in this present study (Zajitschek et al., 2014). However, the association between flagellum length and swimming velocity at the intraspecific level is still debated and varies across species. In the externally fertilizing sea urchin *Heliocidaris erythrogramma* for example, flagellum length is positively correlated with swimming velocity (Fitzpatrick, Garcia‐Gonzalez, & Evans, 2010), and in the internally fertilizing zebra finch *Taeniopygia guttata*, a longer flagellum seems to be beneficial up to a certain point beyond which it seems to slow sperm down again (Bennison et al., 2016). In house sparrows *Passer domesticus*, different studies reached variable conclusions, with one study showing that flagellum length correlates negatively with sperm swimming speed (Cramer et al., 2015) and a different study showing that the relative length of the flagellum correlates positively with swimming speed (Helfenstein, Podevin, & Richner, 2010). In sand martins *Riparia riparia*, sperm length was negatively correlated with swimming speed but positively correlated with sperm longevity (Helfenstein, Szép, Nagy, Kempenaers, & Wagner, 2008), and in tree swallows *Tachycineta bicolor*, no significant association between flagellum length and sperm swimming speed was found (Laskemoen et al., 2010). Similarly, in New World blackbirds (Icteridae), no significant correlation between flagellum length and sperm velocity was found (Lüpold, Linz, & Birkhead, 2009). The size of the midpiece, on the other hand, seems to be the main predictor for sperm swimming velocity in the red deer *Cervus elaphus* (Malo et al., 2006) and the house mouse *Mus musculus* (Firman & Simmons, 2010), and the relative length of midpiece to flagellum was identified as the main predictor in the house sparrow *Passer domesticus* (Helfenstein et al., 2010). However, another study looking at nine intraspecific datasets found no evidence for the relationship between the length of either trait and sperm swimming velocity (Humphries, Evans, & Simmons, 2008). It is currently unclear whether a larger midpiece means more mitochondria and therefore higher efficiency. A study in the Atlantic salmon *Salmo salar* reports higher ATP production by sperm with a longer midpiece (Vladić, Afzelius, & Bronnikov, 2002), whereas a recent study in the zebra finch found that ATP production by sperm with shorter midpieces was higher (Bennison et al., 2016). Finally, a slender head intuitively fits the predictions by physics and biomechanics to be more dynamic even at the micro‐scale of sperm (Humphries et al., 2008). Overall, our current understanding of how morphometric sperm traits contribute to swimming velocity is still far from understood and these associations will likely vary across species, requiring further research.

### 4.2 Physiology

After ejaculation, sperm are exposed to environmental conditions that are very different from those encountered in the male reproductive tract. These conditions are known to affect the sperm performance substantially. In externally fertilizing fish, the contact with water and the change in osmolality is known to activate sperm (Alavi & Cosson, 2006). But the change in osmolality presents a challenge for sperm to resist to. Hypo‐osmotic conditions, as encountered in fresh water environments, cause the take up of water and change the physiological conditions in the different sperm organelles (Alavi & Cosson, 2006; Gwo, 1995). Our results suggest that males may produce sperm that exhibit different levels of resistance to osmotic stress. We found that sperm head shape, midpiece size and flagellum conformation changed more rapidly upon contact with water in sperm from males exposed to low competition. These differences in physiology may affect sperm function and by that their ability to fertilize eggs. In the northern pike *Esox lucius,* for example, the conformation of sperm flagellum was associated with sperm mobility and is affected by the osmolality of the medium (Alavi et al., 2009). Osmotic stress has also been described to affect the integrity of the mitochondria in three marine fish, black porgy *Acanthopagrus schlegelli*, black grouper *Epinephelus malabaricus*, and Atlantic croaker *Micropogonias undulatus*, with size and number of mitochondria decreasing following sperm activation (Gwo, 1995). Our results may provide a possible mechanism underling the recent findings in the Chinook salmon *Oncorhynchus tshawytscha*, where sperm velocity changes in response to variation in the composition of the seminal fluid of males exposed to different levels of sperm competition (Bartlett et al., 2017). Similarly, the effects of the seminal fluid on sperm from males assuming different mating tactics described in two marine goby species (Locatello et al., 2013) could reflect differential osmotic effects on the sperm types produced by different males. Finally, the effect of the ovarian fluid on sperm swimming patterns in externally fertilizing species such as the Arctic charr *Salvelinus alpinus* (Urbach et al., 2005) could be the result of changes in sperm physiology, which ultimately affect their performance.

### 4.3 DNA integrity

The changes in morphometry and physiology may affect the fertilization success of the respective sperm and with that the fitness of the male. In contrast, factors that change the sperm content may be carried over into the next generation and affect the fitness of the offspring. The most dramatic effects are those affecting the genome directly, including the double‐strand breaks we tested for in this study. We found these to be more frequent in sperm from males exposed to high competition. Double‐strand breaks are the most extreme form of DNA damage, and other more subtle effects undetected by the TUNEL assay are likely to be present (Ribeiro, Muratori, Geyter, & Geyter, 2017). A possible explanation for the difference in DNA damage between treatments is that males in the high competition treatment experience higher levels of stress, which have been associated with an increase in double‐strand breaks in humans (Black, Bot, Révész, Scheffer, & Penninx, 2017). The increased production of reactive oxygen species (ROS) in response to physiological stress may explain such an increase in DNA damage as the activity of ROS such as H_2_O_2_ may induce double‐strand breaks in human sperm (Aitken & De Iuliis, 2010; Li, Yang, & Huang, 2006). Negative effects of stress on gamete production have also been reported in the rainbow trout *Onchorhynchus mykiss* (Campbell, Pottinger, & Sumpter, 1992), where repeated exposure to stress not only reduced the sperm count in the males but also the survival in the offspring. Our results might therefore explain the reduced survival of the offspring sired by males under high competition that was reported in a previous study (Zajitschek et al., 2014) using the same experimental set‐up. Whether DNA damage such as double‐strand breaks affects sperm fertilization ability in the zebrafish is currently unclear. However in the house mouse *Mus musculus*, sperm reaching the fallopian tube showed lower levels of DNA fragmentation (Hourcade, Pérez‐Crespo, Fernández‐González, Pintado, & Gutiérrez‐Adán, 2010) and in boar, chromatin‐unstable sperm were less likely to reach oocytes *in vivo* (Ardon et al., 2008). However, it is still unknown if sperm genome integrity can affect the sperm's ability to fertilize eggs. It is possible that sperm with compromised DNA integrity are selected against before fertilization. This would require a different explanation for the previously observed difference in offspring mortality. Beside the DNA, other components of the sperm head content such as RNAs, epigenetic marks and proteins (Immler, 2018) may also be affected, but we did not look at these. Future studies are warranted to further investigate the effect of social conditions on other sperm factors.

Another observation in our study was the increase in number of cells signalling DNA damage over time after activation. These results are in line with a previous study where similar cell damage was observed during sperm activation in zebrafish (Gosálvez, López‐Fernández, Hermoso, Fernández, & Kjelland, 2014). Sperm DNA damage can be caused by oxidative stress during activation (Muratori et al., 2015). The main increase occurred during the first two minutes post‐activation, which is the biologically relevant time for zebrafish sperm. Fertilization takes place within seconds after gamete release but sperm may swim actively for up to two minutes (Coward, Bromage, Hibbitt, & Parrington, 2002). Such an increase in DNA damage suggests that as time passes post‐activation, offspring should suffer from increasingly reduced survival. However, the opposite has been described in the zebrafish, where a delay between sperm activation and fertilization by 25 s increases offspring survival (Alavioon et al., 2017). The effects of post‐ejaculation sperm ageing however have been described in many other species where the timing of ejaculation and fertilization may lay further apart (see Reinhardt, 2007; Pizzari et al., 2008 for reviews). The current explanation for the findings of increased offspring fitness in response to selection for longer‐lived sperm in the zebrafish is a link between sperm genotype and sperm phenotype (Alavioon et al., 2017). However, the nature of this link and the genes involved is still not fully understood.

## 5 CONCLUSIONS

Overall, our study provides new insights into how male condition in response to social environment may affect sperm traits. Most importantly, our results suggest that while some traits, such as a longer flagellum, a larger midpiece and a more slender head, may be beneficial for the male by improving sperm performance during sperm competition, the sperm genome may suffer and negatively affect the resulting offspring. In fact, sperm competition could be a driving force favouring the production of better swimmers, potentially at the cost of reduced genetic quality. The potential trade‐off between phenotypic and genetic quality of sperm needs further investigation, and it will be interesting to assess these effects also in other taxa to test for the generality of our findings.

## COMPETING INTERESTS

The authors declare that they have no conflict of interest.

## AUTHOR CONTRIBUTIONS

WTAFS and PSE developed the technical design to assess sperm quality and performed the experiments; WTAFS, PSE, CD and MD collected the data; WTAFS, PSE, STB, AR and SI performed data analyses; and MJGT and SI conceived the experimental design. All authors contributed to the writing of the manuscript.
