# Supplementary material for: The effects of male social environment on sperm phenotype and genome integrity
Source: J Evol Biol. 2019 Mar 25;32(6):535–44. doi: 10.1111/jeb.13435 (PMC6850410; doi:10.1111/jeb.13435)
Supplement: Supplementary file 1 [file JEB-32-535-s001.docx]

***Supplementary Material***

***for***

**The effects of male social environment on sperm phenotype and genome integrity**

**Figures**


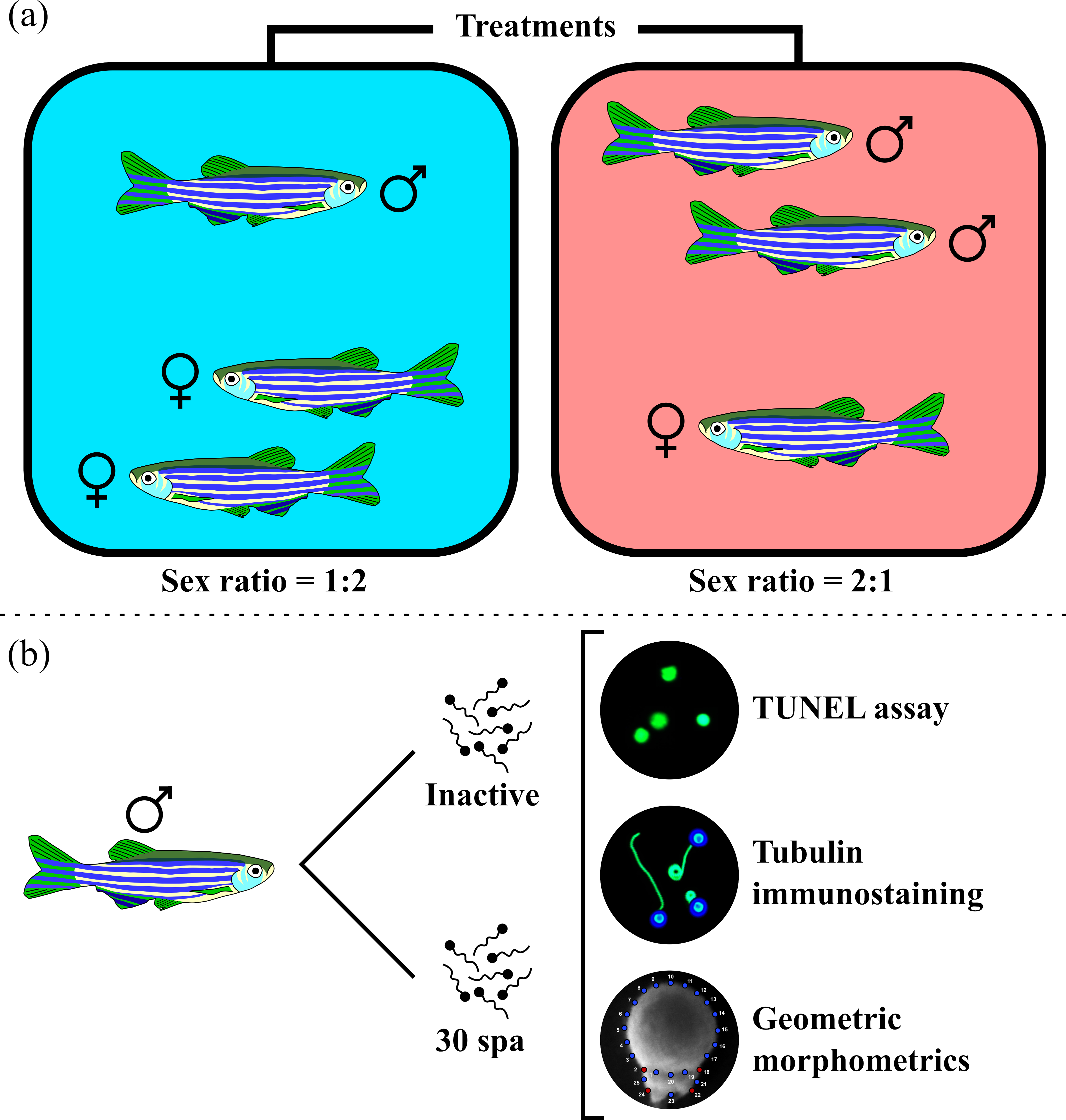
**Figure S1.** Experimental design. (a) Social treatments to which males were exposed: low competition (left) and high competition (right) between males. (b) Methods used to analyze the sperm from experimental males. In addition to inactive and 30 spa (seconds post-activation), the TUNEL assay and the tubulin immunostaining were also applied to later stages (1, 5 and 10 minutes post-activation).


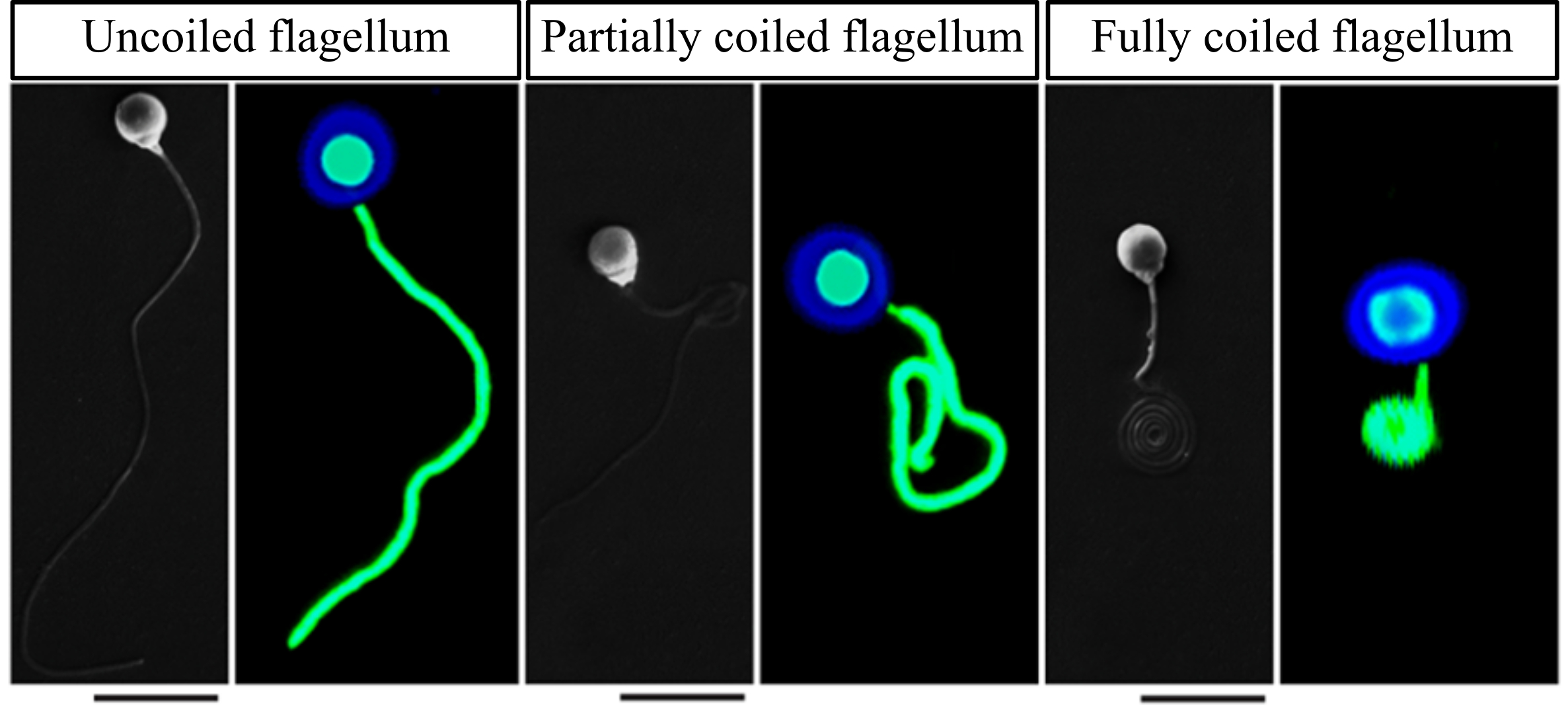
**Figure S2.** SEM and tubulin fluorescence images of zebrafish spermatozoa showing uncoiled, partially coiled and fully coiled flagellum patterns. Scale bars represent 10µm and are common to all images.


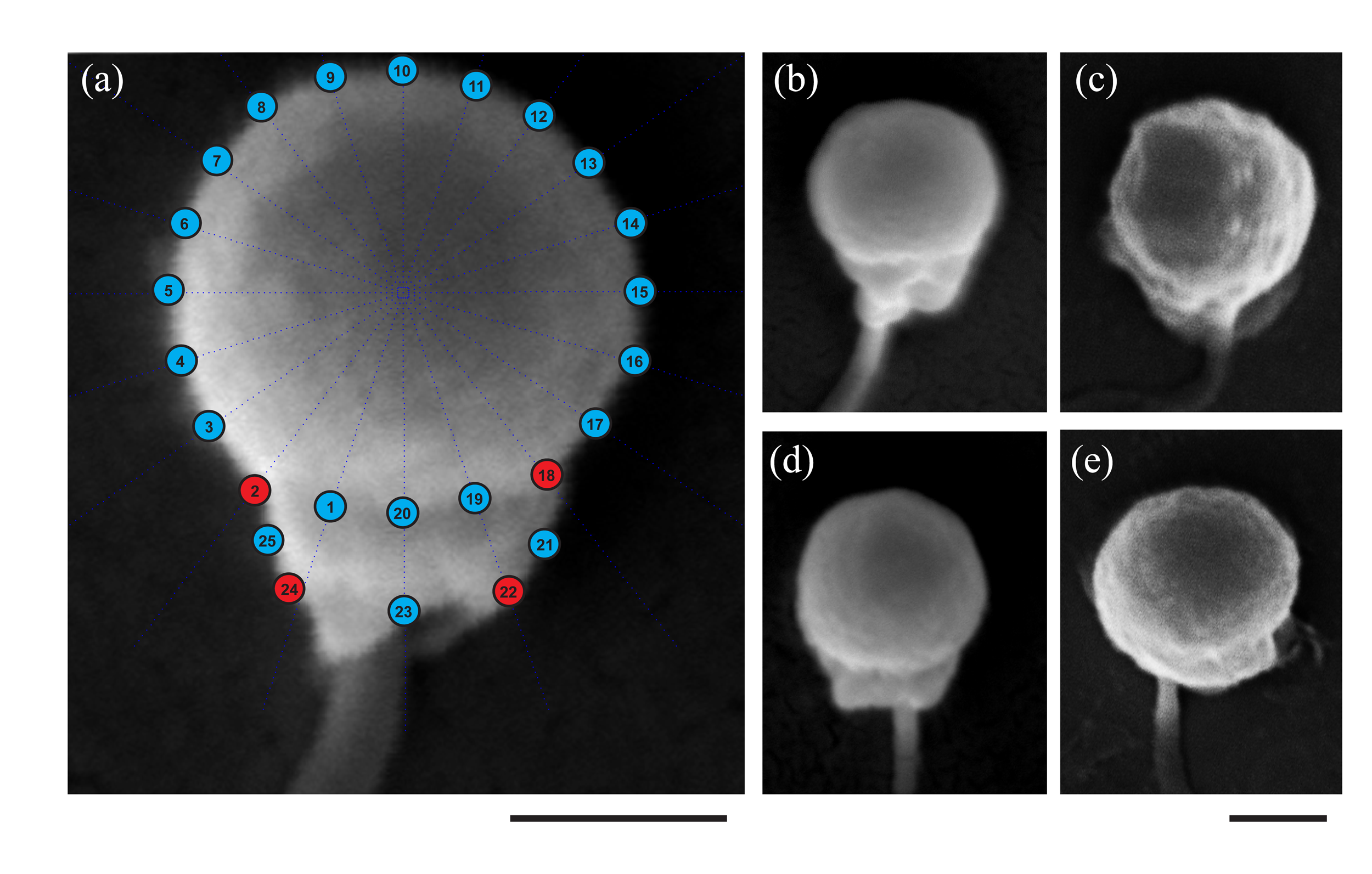


**Figure S3.** Sperm head and midpiece shape analysis using geometric morphometric methods. (a) Landmark and semilandmarks configuration. Red points were treated as a landmarks (2, 18, 22 and 24) and blue points as semilandmarks (1, 3-17, 19-21, 23 and 25). Landmarks 2 and 18 represent identifiable points at the intersection of the midpiece structure with the sperm head. Landmarks 22 and 24 register the points of maximum curvature on the basal side of the sperm midpiece. MakeFan6 (Sheets, 2001) was used to draw 20 equidistant fan lines after locating the head centroid based on two specified endpoints (coordinates 10 and 20). Sperm head semilandmarks (1, 3−17, and 19-20) were defined at the point where the fan line crossed the sperm head outline. The landmarks 22 and 24 were register in most cases with the fan lines traced. In order to define the overall midpiece structure, semilandmarks 21 and 25 were placed between fixed landmarks (2, 24, 18 and 21) and, semilandmark 23 was located at the middle point of the midpiece distal outline directly opposite to the semilandmark 20. Landmarks and semilandmarks were digitized using tpsDig2 (Rohlf, 2015). Semilandmarks were slid to minimize the bending energy of the thin-plate-spline interpolation between each specimen and the Procrustes consensus configuration. This procedure removes the influence of the arbitrary spacing of the semilandmarks and establishes a geometric homology of the semilandmark coordinates within the sample (Gunz & Mitteroecker, 2013). Examples of SEM sperm cells corresponding to each experimental treatment. Inactive high competition (b); 30 spa high competition (c); inactive low competition (d); 30 spa low competition (e). Scale bars represent 1µm and are common to all micrographs.


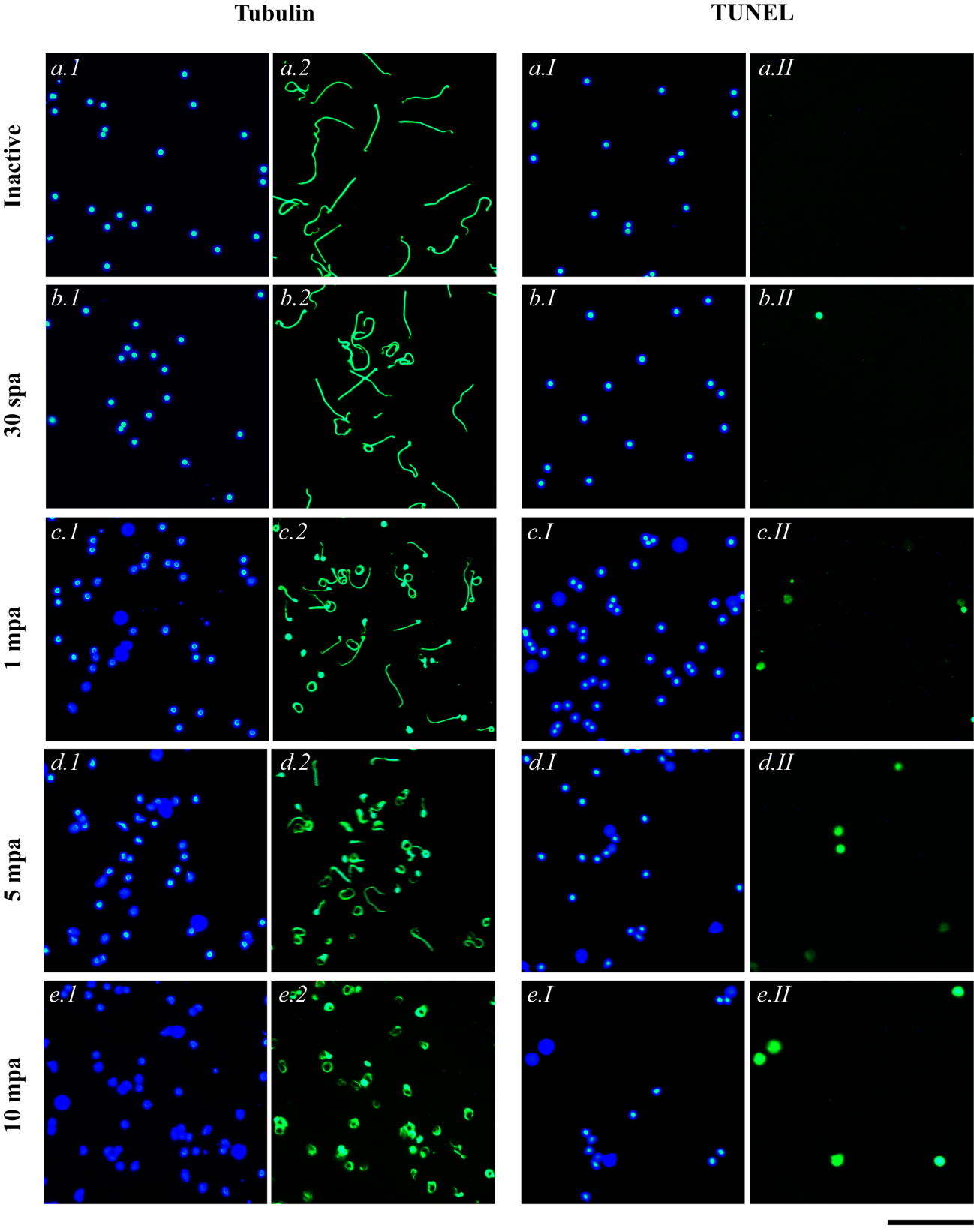


**Figure S4.** Changes in sperm tail conformation (tubulin staining) and TUNEL signal before and after sperm activation as observed under the confocal microscope. (*a.1*-*e.2*) DAPI (blue) and flagellum tubulin (green). (*a.I*-*e.II*) DAPI (blue) and cells with DNA fragmentation (green). Scale bar 50µm common to all images.
